# ISOLATED METACARPAL BONE TUBERCULOSIS-A CASE REPORT

**DOI:** 10.4103/0970-2113.44132

**Published:** 2008

**Authors:** R.A.S Kushwaha, Surya Kant, Sanjay Kumar Verma, Sanjay, Sumit Mehra

**Affiliations:** Department of Pulmonary Medicine, King George's Medical University, Lucknow (India)-226003

## Abstract

**SUMMARY:**

Tuberculous involvement of the metacarpals and phalanges is a rare presentation of extrapulmonary tuberculosis in adult. Here is a case of tubercular dactylitis in a 27 year old female presenting as discharging sinus over proximal part of third metacarpalbone of left hand.

## INTRODUCTION

The spine is the most frequent site of skeletal involvement; occurring in 1 to 3% of patients with extrapulmonary tuberculosis^1^. Tuberculous infection of metacarpals, metatarsal and phalanges of hands and feet is known as tubercular dactylitis. 85% of patients with tubercular dactylitis are younger than 6 years of age^2^. Tubercular dactylitis in adults is rare^3^–^5^.

## CASE REPORT

A 27 year old female patient came to our department with discharging sinus from proximal part of third metacarpal of left hand and pain. Detailed history revealed that she got minor abrasions by some trivial injury over left hand about 2 months back. These abrasions resolved spontaneously but small nodular swelling developed over the proximal part of third metacarpal bone of left hand. During this period she also had pain at local area and low grade pyrexia. She was given antibiotics and other supportive treatment for one month. After one month there was no improvement and sinus formed with discharging pus in that swelling. There was no past history of antitubercular treatment as well as no history of contact to a known case of tuberculosis.

**Fig 1:**
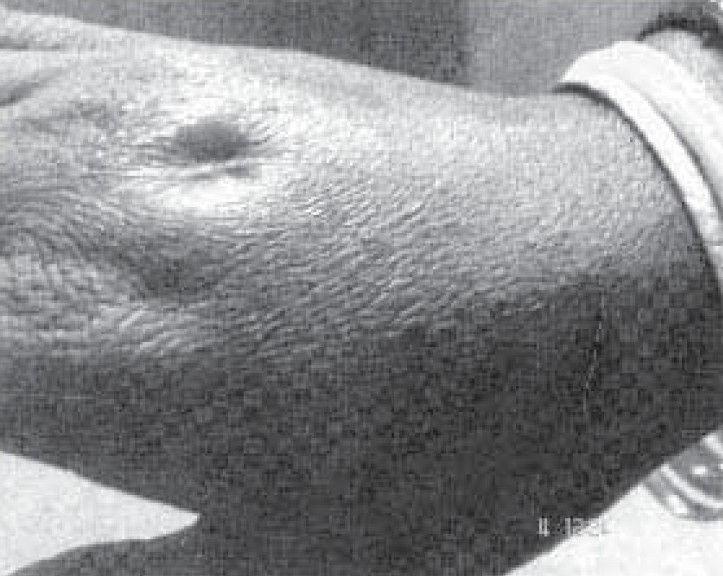
Left hand showing retraction of overlying skin over the proximal part of third metacarpal with pus discharging sinus.

**Fig 2:**
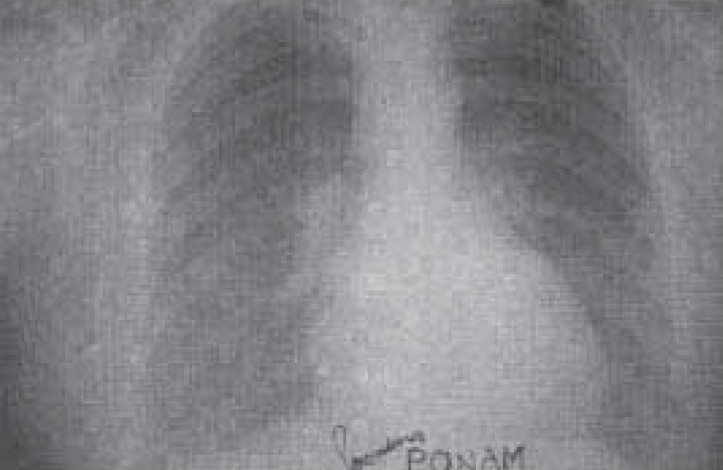
Showing Chest X-ray PA view of patient.

**Fig 3:**
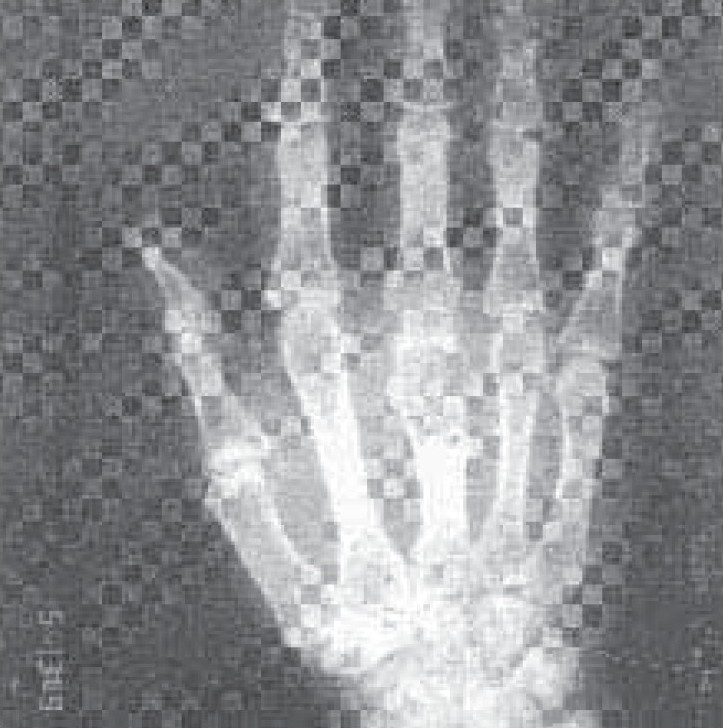
X-ray Left wrist and hand showing lytic lesions over head of third metacarpal bone.

General examination revealed average built of patient with no significant peripheral lymphadenopathy. Examination of left hand revealed retraction of overlying skin over the proximal part of third metacarpal bone of left hand and pus discharging sinus (figure: 1). Her resting pulse rate was 92/min and blood pressure was 112/68mmHg. Respiratory system examination was within normal limit. Examination of others system was unremarkable. Her Haemoglobin was 14 gm%; Total Leucocyte count was 8,900/cmm; Differential Leucocyte count was Neutrophils 72%, Lymphocytes 17% and Monocytes 7%, eosinophil 4% and Erythrocyte sedimentation rate was 58 mm/hour. Her blood culture was sterile. She was HIV seronegative. Her chest x-ray was normal (Figure: 2). X-ray left hand AP and lateral view revealed osteolytic lesions over neck of third metacarpal bone (fig 3). Her Ultrasonography abdomen revealed no abnormality. Her mantoux test showed 34 mm indurations at 72 hours. Pus from discharging sinus was negative for acid fast bacilli on three occasions. Pus culture was positive for Mycobacterium Tuberculosis by Bactec method.

**Fig 4:**
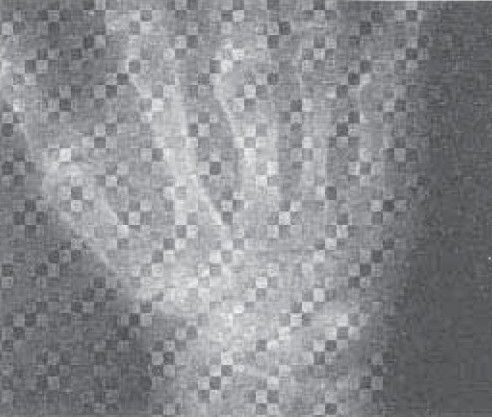
X-ray Left wrist and hand showing less osteolytic activity than previous film.

So the diagnosis of tubercular dactylitis was established and her treatment was began with 4 drugs (Rifampicin, Ethambutol, Isoniazid and Pyrazinamide) for 2 months, followed by 2 drugs (Rifampicin, Isoniazid) for 4 months. Swelling disappeared and sinus healed after six months of institution of antitubercular treatment along with decrement of shadow in radiology (Figure: 4).

## DISCUSSION

Osteoarticular involvement occurs in 1 to 3% of patients with extrapulmonary tuberculosis^1^ and spine represents 50% of these lesions^6^. Tuberculous involvement of the metacarpals and phalanges is a rare presentation of extrapulmonary tuberculosis^3^. 85% of patients with Tubercular dactylitis are younger than 6 years of age^2^. Tubercular dactylitis in adult is rare^3^–^4^. Only 1/3rd of patients with tuberculosis of the bone are diagnosed with concomitant active pulmonary disease^7^. Disseminated skeletal tuberculosis without primary foci is rare^8^. The skeletal infection often becomes symptomatic within 1-3 years after initial infection.

Diagnosis of tubercular dactylitis is made on radiographic features and culture of Mycobacterium Tuberculosis. Radiologically the affected bone appears expanded with lytic lesions in the middle (as seen in present case) and sub periosteal new bone formation along the involved bone. The cavity may contain soft coke like sequestra^9^–^10^. Other findings on plain radiographs include osteopenia, soft-tissue swelling with minimal periosteal reaction, narrowing of joint space, cysts in bone adjacent to joint, and subchondral erosions. The non-specific nature of these radiographic findings can often delay the diagnosis.

The gold standard for the diagnosis of osseous tuberculosis is culture of Mycobacterium tuberculosis from bone tissue^11^. Differential diagnostic considerations include pyogenic osteomyelitis, Brodie's abscess, Kaposi's sarcoma and luetic dactylitis. Clinically, pyogenic osteomyelitis tends to be acutely painful, swollen, and hot with fever. Tuberculous osteomyelitis is relatively benign with mild pain and minimal pyrexia.

Management is essentially by antitubercular drugs, rest to the involved part in functioning position and early active exercise. Current recommendations for the treatment of osseous tuberculosis include a 2-month initial phase of isoniazid, rifampin, pyrazinamide, and ethambutol followed by a 6 to 12-month regimen of isoniazid and rifampin^12^. Few studies argue that 6-month of antitubercular treatment is appropriate for tubercular dactylitis because of its paucibacillary nature.^13^

Tuberculous dactylitis is difficult to diagnose during early stages. Tubercular dactylitis should be suspected in cases of long-standing pain and swelling in the metacarpals and phalanges. It is necessary to keep tubercular dactylitis in mind while making the differential diagnosis of several osseous pathologies.
